# Case report: Beneficial effects of visual cortex tDCS stimulation combined with visual training in patients with visual field defects

**DOI:** 10.3389/fneur.2024.1344348

**Published:** 2024-01-24

**Authors:** Yanhua Lian, Xiaoping Cheng, Qunlin Chen, Libin Huang, Lili Xie, Wenzong Wang, Jun Ni, Xinyuan Chen

**Affiliations:** ^1^Department of Rehabilitation Medicine, The First Affiliated Hospital of Fujian Medical University, Fuzhou, China; ^2^Department of Rehabilitation, Fuzhou Second Hospital, Fuzhou, China; ^3^Department of Radiology, The First Affiliated Hospital of Fujian Medical University, Fuzhou, China; ^4^Department of Ophthalmology, The First Affiliated Hospital of Fujian Medical University, Fuzhou, China; ^5^Department of Rehabilitation Medicine, National Regional Medical Center, Binhai Campus of the First Affiliated Hospital, Fujian Medical University, Fuzhou, China

**Keywords:** visual field defect, transcranial direct current stimulation, visual recovery strategies, perceptual training, case report

## Abstract

**Background:**

Visual field defect (VFD) refers to the phenomenon that the eye is unable to see a certain area within the normal range of vision, which may be caused by eye diseases, neurological diseases and other reasons. Transcranial direct current stimulation (tDCS) is expected to be an effective treatment for the recovery or partial recovery of VFD. This paper describes the potential for tDCS in combination with visual retraining strategies to have a positive impact on vision recovery, and the potential for neuroplasticity to play a key role in vision recovery.

**Methods:**

This case report includes two patients. Patient 1 was diagnosed with a right occipital hemorrhage and homonymous hemianopia. Patient 2 had multiple facial fractures, a contusion of the right eye, and damage to the optic nerve of the right eye, which was diagnosed as a peripheral nerve injury (optic nerve injury). We administered a series of treatments to two patients, including transcranial direct current stimulation; visual field restoration rehabilitation: paracentric gaze training, upper and lower visual field training, VR rehabilitation, and perceptual training. One time per day, 5 days per week, total 6 weeks.

**Results:**

After 6 weeks of visual rehabilitation and tDCS treatment, Patient 1 Humphrey visual field examination showed a significant improvement compared to the initial visit, with a reduction in the extent of visual field defects, increased visual acuity, and improvement in most visual functions. Patient 2 had an expanded visual field, improved visual sensitivity, and substantial improvement in visual function.

**Conclusion:**

Our case reports support the feasibility and effectiveness of tDCS combined with visual rehabilitation training in the treatment of occipital stroke and optic nerve injury settings.

## Introduction

Visual field defect (VFD) is an impairment of the extent of the visual field (VF) caused by injury to the retina, optic nerve, or brain. Most patients with VFDs are partially blind, rather than completely blind (1). It is not possible to completely normalize a VFD, but the brain can amplify the residual signal through neuroplastic mechanisms, so recovery or partial recovery is possible (2). Transcranial direct current stimulation (tDCS), a non-invasive brain stimulation technique, uses electrodes to apply a constant, low-intensity direct current (1–2 mA) to a specific brain region that modulates the neural activity of the cerebral cortex, thereby altering the plasticity of the stimulated brain region. Anodal tDCS increases cortical excitability beneath the electrodes and cathodal tDCS decreases cortical excitability. tDCS is considered a promising therapeutic tool to restore synaptic plasticity in damaged cortices (3). However, its relevant principles and mechanisms of action are not yet clear, and applications of tDCS in clinical diseases are still being explored.

A relatively small number of studies have applied tDCS to treat different causes of VFDs (4), possibly through enhanced visual cortical reorganization, altered neuronal excitability, or increased brain plasticity to produce better results. Räty et al. explored different brain stimulation modalities to treat chronic post-stroke homonymous hemianopia, and only the tDCS modality improved visual acuity (VA) (5). In an observational study by Fujikado et al. (6), electrical stimulation (ES) was performed in the eyes of five patients with traumatic optic neuropathy, and two patients had increased peripheral VF area. Visual perception is affected by transcranial ES (tES) through a complex interaction between stimulus intensity and cortical anatomy (7). However, combining vision training with non-invasive brain current stimulation seems to be more effective in early recovery of visual nerve component damage caused by stroke (8).

Based on previous research, we attempted to treat two patients with VFDs of different causes using tDCS combined with visual recovery strategies, oculomotor compensatory strategies, and perceptual training. These actions resulted in significant improvements in VFs of both patients and allow us to speculate on the possible underlying mechanisms.

## Case description

**Figure 1:**
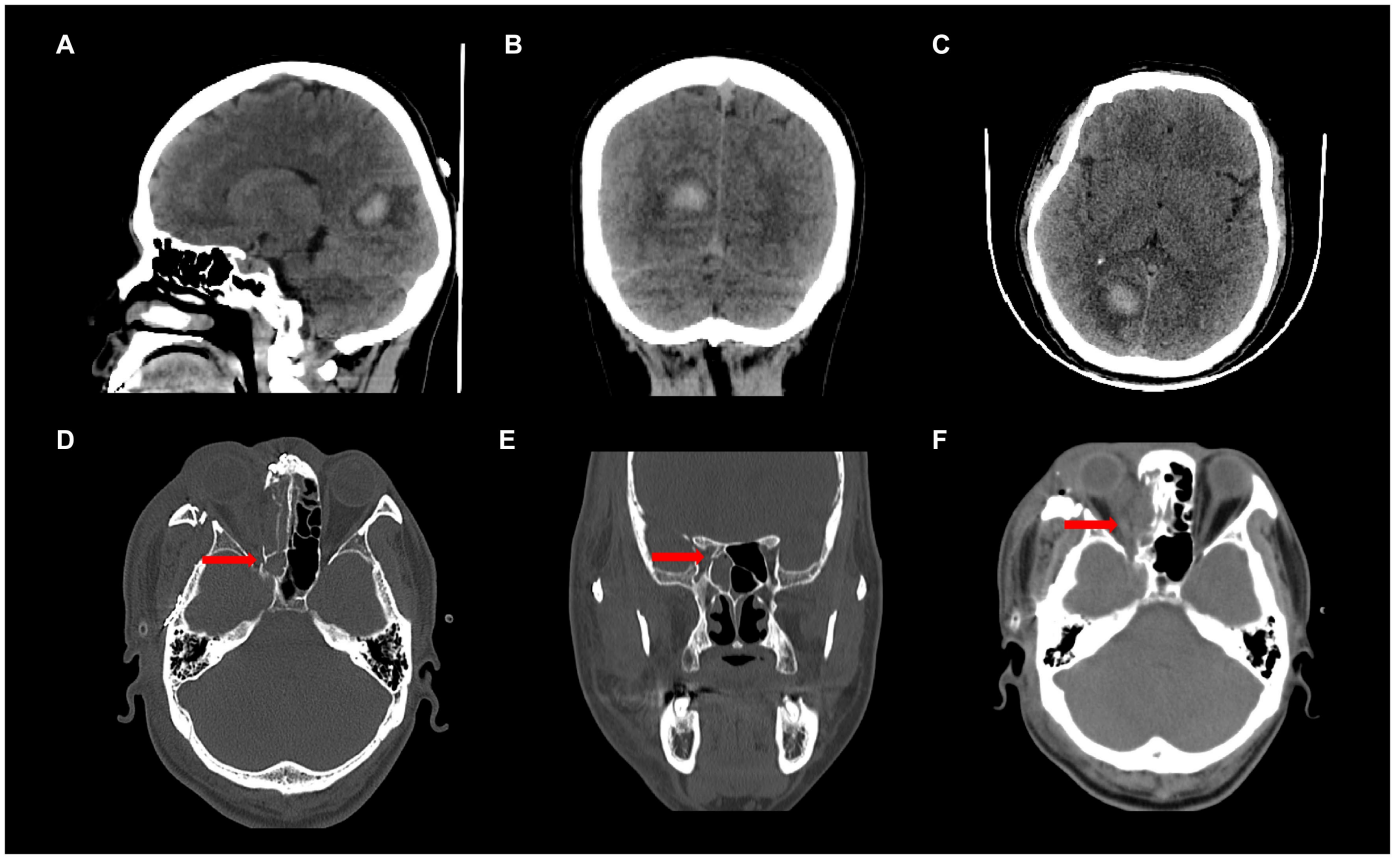
**(A-C)** Patient 1, CT findings showing right occipital lobe hemorrhage; **(D-F)** Patient 2, CT findings showing a fracture of the optic canal and swelling of the optic nerve on the right side.

Patient 1 was male, 34 years old (Table 1), and was found to have VF abnormalities in the right eye on Humphrey visual field examination: VFI (visual field index), 73%; MD (mean deviation), −15.45 dB; PSD (pattern standard deviation), 13.31 dB; and in the left eye: VFI, 76%; MD, −12.87 dB; PSD, 13.32 dB; which was diagnosed as “homonymous hemianopia.” The patient was then diagnosed with a right occipital hemorrhage and operated on (Figures 1A–C). Postoperative CT results showed good absorption of the occipital hemorrhage, and the patient consulted our outpatient clinic, but showed no further significant improvement of the VFD. Admission to our hospital showed a normal physical examination, and the patient demonstrated normal cognition, with bilateral pupils large and equal circles, a sensitive light reflex, and normal movements of the bilateral eye.

**Table 1:** Basic information about the two patients.

Case	Age	Sex	Diagnosis	Surgeries
1	34	Male	Right occipital hemorrhage(homonymous hemianopia)	Removal of right occipital haematoma, excision of malformed vascular mass in right occipital lobe
2	46	Male	Multiple facial bone fractures (contusion of the right eye)	Right eyelid, root of the nose, zygomatic clearing and suturing

Patient 2 was male, 46 years old (Table 1), and had multiple facial bone fractures (of the right orbitozygomatic maxilla, frontal bone, nasal bone, pterygoid bone, and sieve bone), contusion of the right eye, and damage to the optic nerve of the right eye (Figures 1D–F) caused by an impact of explosive debris to his head during operation of a fishing vessel. Postoperative MRI findings showed abnormal right eye position, a distorted optic nerve, and a dilated right superior ophthalmic vein. Postoperative examination showed that the pupils were unequal in size and roundness bilaterally, the right eye had extremely weak vision, subconjunctival hemorrhage and edema, corneal edema, and the right pupil was about 7 mm in diameter, the response to light was absent, and eye movements were uncooperative. The left upper and lower eyelids were cyanotic, the left pupil was about 3 mm in diameter, the eye was sensitive to light, there was no diplopia, no blurring of the VF, and the eye movements were acceptable. Humphrey visual field examination of the right eye showed: VFI, 47%; MD, −21.10 dB; and PSD, 13.58 dB; confirming the diagnosis of peripheral nerve injury (optic nerve injury) resulting in VFDs in the right eye.

## Diagnostic assessment

### Treatment

We treated both patients with a series of interventions (VF restoration rehabilitation: paracentral gaze training, upper and lower VF training, VR rehabilitation and perceptual training), including tDCS with the following stimulation parameters: a sponge electrode immersed in saline-soaked (0.9% NaCl) from two electrodes (5 cm^2^ × 1, 10 cm^2^ × 1) connected to a 9 V battery-driven stimulator (direct current delivery utilized an IS200 portable battery-driven device manufactured by Chengdu, China) to provide a constant current of 1.5 mA for 20 min during training. According to the 10–20 international EEG coordinate system, the anode was placed at the occipital pole (Oz) and the cathode was placed at the chest, then a non-latex rubber belt was used to fix the electrode. Both patients understood the study and gave written consent according to the Declaration of Helsinki (1964), which was approved by the Ethics Committee of the First Hospital of Fujian Medical University (MRCTA, ECFAH of [2015]084).

**Figure 2:**
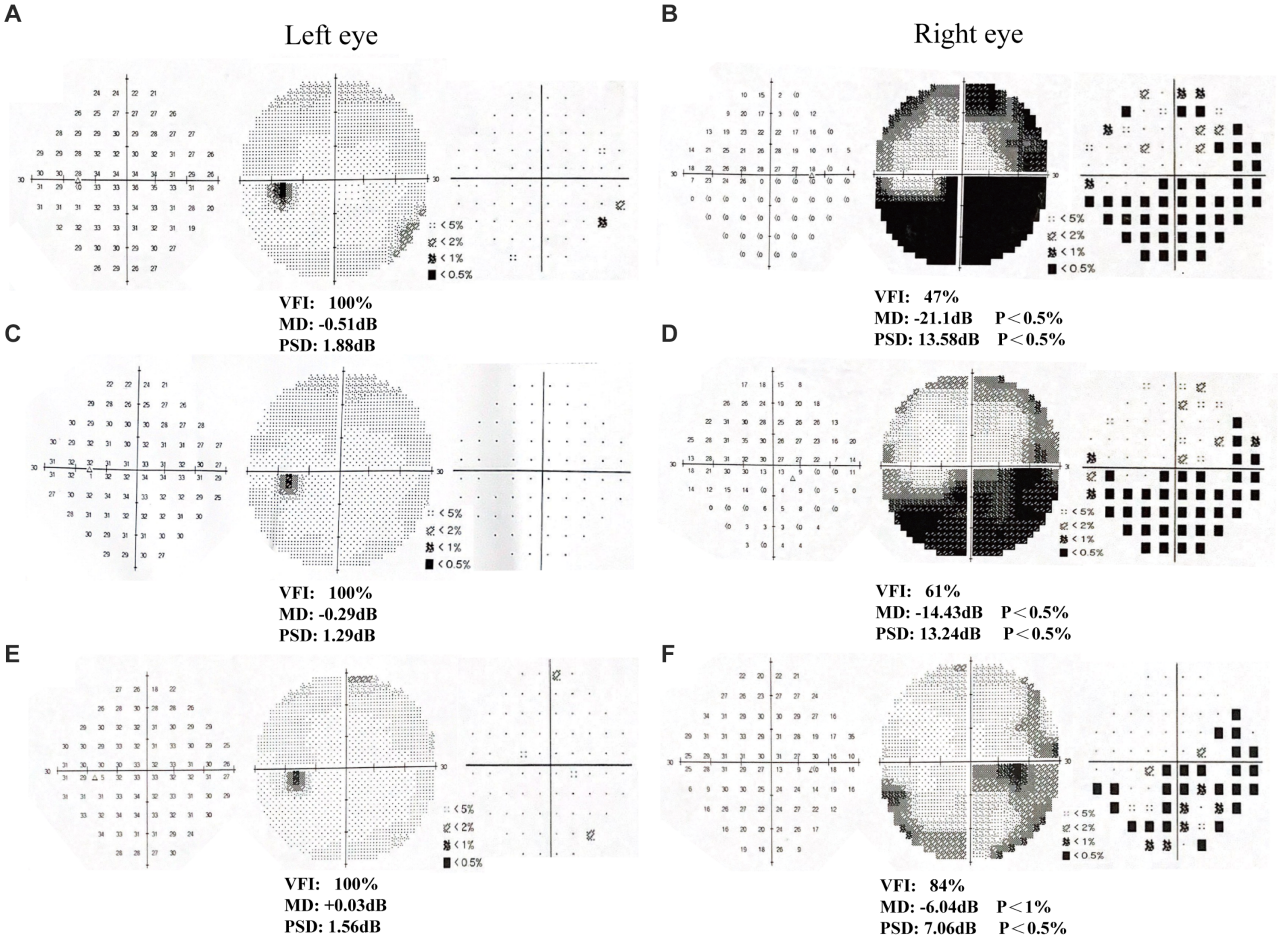
Humphrey visual field examination results in patient 1. **(A-B)** First examination of patient’s binoculus; **(C-D)** Second examination of patient’s binoculus; **(E-F)** Third examination of patient’s binoculus.

**Figure 3:**
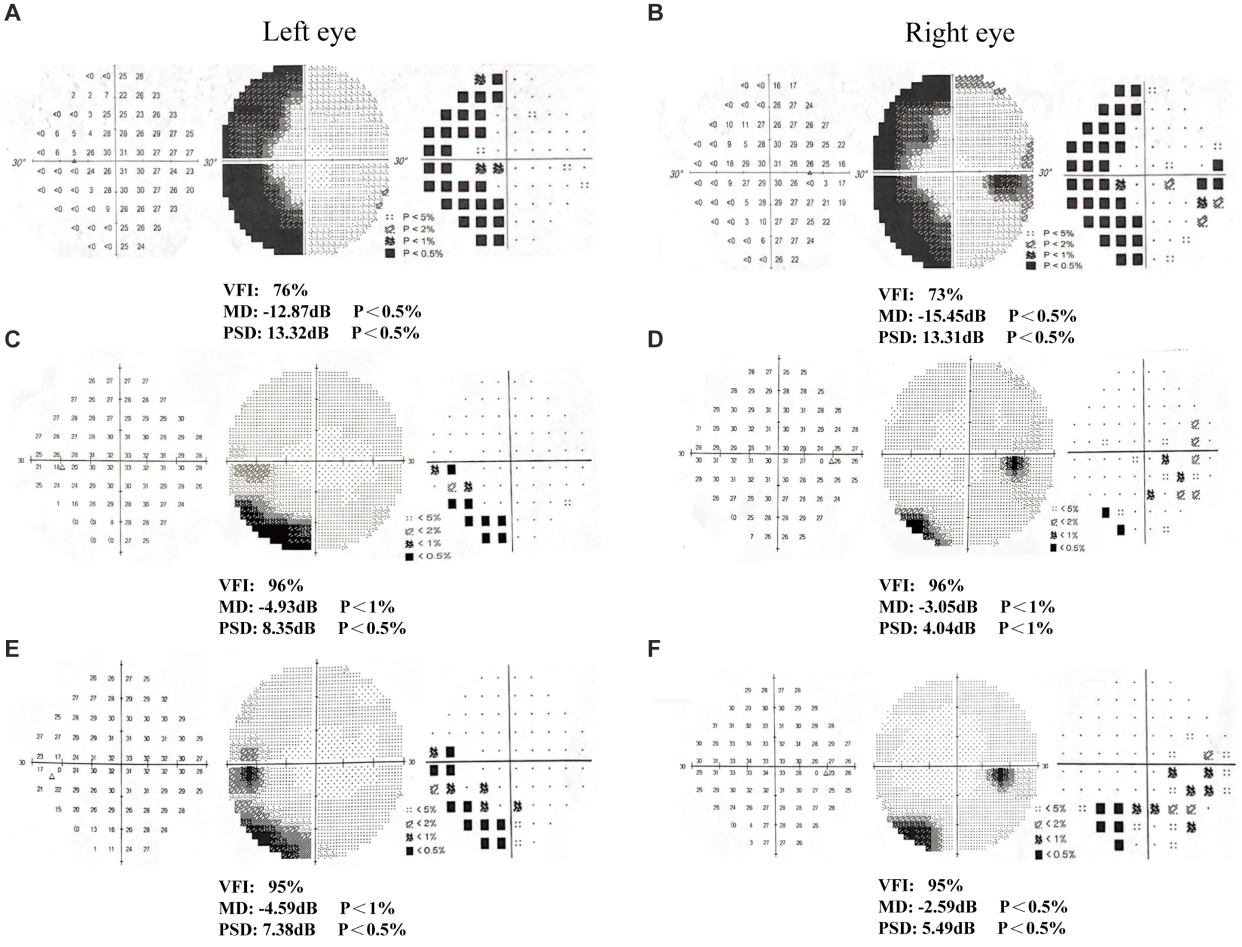
Humphrey visual field examination results in patient 2. **(A-B)** First examination of patient’s binoculus; **(C-D)** Second examination of patient’s binoculus; **(E-F)** Third examination of patient’s binoculus.

After 6 weeks (1 time per day, 5 days per week) of visual rehabilitation training and tDCS treatment, Humphrey VF examination for Patient 1 showed VFI, 95%; MD, −4.59 dB; and PSD, 7.38 dB in the left eye; and VFI, 95%; MD, −2.59 dB; and PSD, 5.49 dB in the right eye, 19% enhancement of VFI in the left eye and 22% improvement in VFI in the right eye compared to first treatment (Table 2 and Figures 2A–F). These results suggested significant improvement compared with the initial visit, with a reduction in the extent of VFDs. VA increased, and most visual functions improved. In Patient 2, the Humphrey VF examination in the right eye showed VFI, 84%; MD, −6.04 dB; and PSD, 7.06 dB, compared with the first treatment, the VFI of the right eye increased by 37%, and the VFI of the left eye as the healthy side remained unchanged (Table 2 and Figures 3A–F). VF range expanded, VA improved, and visual function was substantially improved.

**Table 2:** Results of three-times Humphrey visual field examination in two patients.

	Visual field index				Mean deviation (dB)			Pattern standard deviation (dB)		
	First time	Second time	Third time	percentage of change	First time	Second time	Third time	First time	Second time	Third time
Patient 1 (left eye)	76%	96%	95%	19%	−12.87	−4.93	−4.59	13.32	8.35	7.38
Patient 1 (right eye)	73%	96%	95%	22%	−15.45	−3.05	−2.59	13.31	4.04	5.49
Patient 2 (left eye)	100%	100%	100%	0%	−0.51	− 0.29	0.03	1.88	1.29	1.56
Patient 2 (right eye)	47%	61%	84%	37%	−21.1	−14.43	−6.04	13.58	13. 24	7.06

Percentage of change = VFI (Third time)–VFI (First time).

## Discussion

In the present case report, the extent of VFDs resulting from both central (occipital stroke) and peripheral (optic nerve injury) injuries were reduced after a long course of tDCS combined with conventional rehabilitation training. Previous studies have shown that visual rehabilitation (9) and tDCS treatment (3) can stimulate brain plasticity and positively affect visual function recovery. tDCS applied to visual cortical and peripheral areas can alter visual evoked potentials or affect visual search performance (10). Gall et al. (11) first applied non-invasive current stimulation to treat stroke-associated VFDs, and the results were positive. In stroke patients, tDCS effectively improved visual awareness, motion perception, and contrast discrimination (8). Another noninvasive brain stimulation technique, transcranial alternating current stimulation (tACS), is often used to treat optic neuropathy (12), which improves impaired VA and VF (13). Current treatment mechanisms are based on the following hypotheses: (1) residual visual activation theory, in which visual function improves after excitatory changes in the visual cortex and other brain structures, following non-invasive brain stimulation; (2) reactivation and restoration of neural tissue is possible if residual functional structures undergo repeated use (1); and (3) reorganization of the brain’s functional connectivity network (1). Due to the above hypotheses, we treated these two patients with visual deficits with a long course of treatment using the protocol of occipital tDCS, along with VF restoration rehabilitation training and achieved good results.

Occipital stroke is a common cause of VF loss (14), but the underlying mechanism for the reorganization of visually relevant cortical networks as a possible cause of VFD recovery after stroke remains unclear. Gamma-aminobutyric acid (GABA) transmission is inhibited in the cerebral cortex by enhanced excitatory synaptic transmission brought on by anodic tDCS stimulation, which may favor glutamatergic transmission by changing the balance of GABA and glutamatergic activity. (15–17). We hypothesize that tDCS enhances the visual training effect by modulating the excitability of surviving visual networks to improve VFDs caused by visual cortical damage, or by influencing neurotransmitter systems, such as GABA (16), thereby mediating changes in neuronal excitability (18), and promoting activation of visual pathways. Evidence suggests that tDCS modulates cortical excitability by facilitating cortical connectivity (19) and changing the membrane potential of neurons. The results of Cabib et al. point to the role of tDCS in enhancing brainstem excitability, with the facilitation of cortical downstream pathways as a possible mechanism (20). Bolzoni et al. (21) demonstrated activation of subcortical motor system neurons after transcranial brain stimulation that was polarized. tDCS continues afferent to the occipital cortex, affects other cortical areas through the cortical network, and transmits excitability to the optic pathway to, respectively, restore or activate the optic nerve or remaining undamaged nerves, facilitating VF recovery. Both Alber et al. (8) and Plow (22) showed that tDCS has a facilitative effect in treating partial blindness when associated with training. Compared with visual rehabilitation training alone, the combination of occipital tDCS and VF rehabilitation training has a facilitative effect on visual function (8). In addition, visual rehabilitation-related training can stimulate brain plasticity (9).

First, due to the case study nature of this study, we were unable to conduct a non-blinded intervention and lacked a control intervention. Second, we currently combined tDCS with brain oculomotor compensation, visual restoration, and perceptual training, and therefore could not discern the effects of each of the different therapies. Therefore, further studies in large populations are needed to validate effects.

## Conclusion

Preliminary case results demonstrate the feasibility and efficacy of tDCS combined with visual rehabilitation training for the treatment of occipital stroke and optic nerve injury. tDCS may modulate the excitability of surviving visual networks and promote VF recovery through changes in neuronal excitability or induce peripheral neuroplasticity by stimulating the central modulation of brain plasticity, which activates the visual pathway and improves VFD.

## Data availability statement

The datasets presented in this article are not readily available because of ethical and privacy restrictions. Requests to access the datasets should be directed to the corresponding authors.

## Ethics statement

The studies involving humans were approved by the Ethics Committee of the First Hospital of Fujian Medical University. The studies were conducted in accordance with the local legislation and institutional requirements. The participants provided their written informed consent to participate in this study. Written informed consent was obtained from the individual(s) for the publication of any potentially identifiable images or data included in this article.

## Author contributions

YL: Writing – original draft. XiaC: Writing – original draft. QC: Writing – original draft. LH: Writing – original draft. LX: Writing – original draft. WW: Writing – original draft. JN: Writing – review & editing. XinC: Writing – review & editing.
